# Association between proton pump inhibitors and periodontal disease severity

**DOI:** 10.1002/cre2.495

**Published:** 2021-09-21

**Authors:** Bhavneet K. Chawla, Robert E. Cohen, Lisa M. Yerke

**Affiliations:** ^1^ Department of Periodontics and Endodontics, School of Dental Medicine, The State University of New York University at Buffalo Buffalo New York USA

**Keywords:** medications, periodontal disease, periodontitis, probing depth, proton pump inhibitors

## Abstract

**Objectives:**

Proton pump inhibitors (PPIs) are commonly prescribed for the management of acid‐related gastrointestinal disorders. PPIs modulate osteoclast function, reduce gastric acid secretion, and are associated with the establishment of a more diverse gastrointestinal microbiota. Periodontitis is characterized by microbe‐associated host‐mediated inflammation that results in loss of periodontal attachment. The aim of this study was to assess whether a relationship exists between PPIs and periodontal disease.

**Materials and methods:**

A retrospective analysis was performed using patient records from a faculty periodontal practice. The proportion of elevated probing depths was used to measure periodontitis severity. Statistical analysis was performed using independent sample *t*‐tests, and Chi‐square tests of independence.

**Results:**

Records from 1093 patients were initially assessed. Fourteen percent of teeth were associated with ≥6 mm probing depths among PPI users, in contrast to 24% for patients not using PPIs (*P* = 0.030). Similarly, 27% of teeth exhibited ≥5 mm probing depths among PPI users versus 40% for non‐PPI users (*P* = 0.039).

**Conclusions:**

The results suggest that PPIs are associated with a reduced proportion of elevated probing depths. Future prospective studies are indicated to elucidate possible mechanisms through which PPIs might affect, and potentially be used in the treatment of, periodontitis.

## 1 INTRODUCTION

Proton pump inhibitors (PPIs) such as omeprazole and pantoprazole are commonly prescribed for the management of acid‐related gastrointestinal (GI) disorders (Richardson et al., 1998). Ranked among the 10 most frequently prescribed drugs in the United States, PPIs are used to treat a variety of conditions, including gastroesophageal reflux disease, peptic ulcers, *Helicobacter pylori* infection, Barrett's esophagus, as well as to treat patients who might be at higher risk for those conditions, such as patients on long‐term nonsteroidal anti‐inflammatory (NSAIDS) or antiplatelet therapies (Brisebois et al., 2018; Rotman & Bishop, 2013; Williams & McColl, 2006).

PPIs inhibit the H^+^/K^+^ adenosine triphosphatase proton pump in gastric parietal cells, inhibiting the secretion of gastric acid into the stomach (Ali et al., 2009). Typically, gastric parietal cells secrete hydrochloric acid when stimulated, and a negative feedback inhibitory mechanism initiated in response to elevated intragastric acidity depresses further acid secretion (Laine et al., 2000). Interruption of that mechanism by drugs such as PPIs results in hypergastrinemia. Chronic low‐acid states have been implicated in the reduced absorption of fats, minerals, and vitamins (Laine et al., 2000).

In addition to aiding in digestion and activating pepsin, gastric acid limits the survival of pH‐sensitive microorganisms implicated in GI tract infections (Clooney et al., 2016). Accordingly, an association has been found between the composition of the microbiome and PPI use (Jackson et al., 2016). PPIs increase stomach pH, which decreases the number of indigenous bacteria and encourages alternative bacterial species to survive and colonize (Imhann et al., 2016). Changes in the GI microbiota with PPI use also has been related to an increased risk of bacterial peritonitis, pneumonia, and enteric infections (Williams & McColl, 2006). Achlorhydria induced by acid‐inhibitory drugs can lead to bacterial overgrowth, consisting primarily of Gram‐positive organisms that more closely resemble the oral and oropharyngeal microflora (Williams & McColl, 2006).

Evidence suggests that PPI administration might have a deleterious effect on developing bone among pediatric patients, and lead to decreased bone mineralization and development of osteoporosis in adult or elderly individuals (Wagner, 2018). However, the precise mechanism by which PPIs impact bone metabolism is unclear. One theory suggests that PPIs might reduce intestinal calcium absorption by increasing the gastric pH, thereby affecting the solubility of calcium salts obtained from the diet (Wagner, 2018). Decreased calcium absorption results in increased risk of bone resorption and secondary hyperparathyroidism, leading to a negative calcium balance (Vestergaard et al., 2006; Yang, 2012). Another theory suggests that suppression of gastric acid by PPIs leads to hypergastrinemia, which has a stimulatory effect on parathyroid glands. Parathyroid hormone (PTH) has been reported to play a pivotal role in calcium and bone metabolism, and increased levels of PTH stimulates bone resorption to maintain the serum calcium concentration (Yang, 2012). Indeed, long‐term elevated PTH levels have been associated with decreased bone strength and quality (Yang, 2012).

Yet another hypothesized mechanism by which PPIs impact bone metabolism is through direct inhibition of bone‐specific proton pumps that are present in osteoclasts (Brisebois et al., 2018). Osteoclasts resorb bone by lowering the pH at their external ruffled border area (Tuukkanen & Vaananen, 1986). Tuukkanen et al., in an in vitro study, found many similarities between gastric acid production and osteoclast‐mediated bone resorption, an observation that supports the concept that the basic cellular event in osteoclast‐mediated bone resorption occurs by active H^+^ production at the site of active resorption (Tuukkanen & Vaananen, 1986).

Periodontitis is characterized by microbe‐associated host‐mediated inflammation that results in loss of periodontal attachment (Tonetti et al., 2018). The pathogenesis of periodontitis can be significantly influenced by the host response, which facilitates the subsequent loss of periodontal attachment initiated by bacteria (Hienz et al., 2015). Once the immunoinflammatory processes begin, the connective tissue attachment and alveolar bone are destroyed and the junctional epithelium migrates apically (Hienz et al., 2015). Bone destruction by osteoclastic resorption, in addition to inflammation, tissue destruction, and detachment of junctional epithelium, often results in formation of periodontal pockets and, ultimately, tooth loss (Bosshardt, 2018; Hienz et al., 2015).

Since PPIs can alter the gut microbiome and affect bone, and the etiology and pathogenesis of periodontal disease are influenced by bacteria within the periodontal pocket, we hypothesized that PPIs might have the ability to affect periodontal pathogenesis. Consequently, the primary objective of this study was to assess the relationship of PPIs and periodontal disease through comparison of the proportion of elevated periodontal probing depths at teeth from patients using or not using PPIs.

## 2 MATERIALS AND METHODS

### 2.1 Study design and patient population

This project was reviewed and approved by the University at Buffalo Health Sciences Institutional Review Board (Study#00001811), which conforms to the ethical standards set by the Declaration of Helsinki and its later amendments. Informed consent was not required for this retrospective study. The manuscript was prepared according to the Strengthening the Reporting of Observational Studies in Epidemiology (STROBE) guidelines (von Elm et al., 2014). Patients 18 years or older, who received evaluation at a faculty periodontal specialty practice from 1996–2015, were identified for initial review.

Demographic information was obtained, as well as periodontal probing depths, plaque scores, PPI status, and medical histories. Periodontal probing depths included measurements from six sites around each tooth (mesiobuccal, midbuccal, distobuccal, mesiolingual/palatal, midlingual/palatal, and distolingual/palatal), excluding third molars. The percentage of teeth having at least one probing depth of ≥5 and ≥ 6 mm was separately calculated for assessment of moderate to severe chronic periodontitis based on guidelines suggested by the Centers for Disease Control and Prevention, and the American Academy of Periodontology (CDC/AAP) criteria for assessment of chronic periodontitis (Page & Eke, 2007). Patient home care was assessed at the time of patient examination using the Silness and Löe plaque index (Fischman, 1986). To reduce bias and inter‐examiner variability, all patient evaluations, as well as clinical measurements, were performed by the same examiner (R.C.). PPI medications included in this study were omeprazole, esomeprazole, lansoprazole, rabeprazole, pantoprazole, and dexlansoprazole.

Records from a total of 1093 patients (≥18 years old) were available for initial consideration for this study. Statistical analysis was performed after excluding smokers and diabetics (NS/ND). To reduce the influence of other potential confounding factors, the analysis was repeated after further excluding systemic factors such as history of chemotherapy, use of systemic steroids or hormone replacement therapy, as well as non‐thyroid autoimmune diseases such as rheumatoid arthritis and systemic lupus erythematosus (NS/ND/NMed).

### 2.2 Statistical analyses

Statistical analysis was performed to measure differences in probing depths between PPI and non‐PPI groups, as well as mean patient age differences in those groups, using independent sample *t*‐tests adjusted for equal or unequal variances as appropriate. Significance was measured via computation of the 95% confidence interval and *P* value of the group mean differences. For plaque score measurements, discontinuous plaque scores were transformed to either low plaque (PI ≤1.5) or high plaque (PI >1.5) categories for PPI and non‐PPI groups, and analyzed using Chi‐square tests of independence. All calculations were performed using IBM SPSS Statistics v26.

## 3 RESULTS

Among 31 PPI users in the NS/ND group, 14% of teeth had probing depths ≥6 mm, in contrast to 24% for the 713 patients not using PPIs (*P* = 0.017). Similarly, 28% of teeth in the NS/ND group exhibited probing depths ≥5 mm among those using PPIs versus 40% for patients not using PPIs (*P* = 0.032; Table 1). There were no significant differences in the mean plaque scores among PPI users and nonusers (*P* = 0.875; Table 2). However, the mean age of PPI users (61 years) was greater than that of nonusers (56 years; *P* = 0.021; Table 3).

**Table 1:** Mean percentage of teeth with elevated periodontal probing depths in patients using PPIs versus not using PPIs

PPD‐PPI	% Of teeth with PPD ≥5 mm (NS/ND)^a^	% Of teeth with PPD ≥6 mm (NS/ND)^a^	% Of teeth with PPD ≥5 mm (NS/ND/NMed)^b^	% Of teeth with PPD ≥6 mm (NS/ND/NMed)^b^
No PPI	40.5% (*N* = 713)	24.2% (*N* = 713)	40% (*N* = 621)	23.7% (*N* = 621)
PPI	27.8% (*N* = 31)	13.9% (*N* = 31)	27.2% (*N* = 28)	14% (*N* = 28)
Mean difference (no PPI‐PPI)	12.7%; 31% decrease	10.3%; 42% decrease	12.8%; 32% decrease	9.7%; 41% decrease
95% Confidence interval of mean difference	0.01–0.24	0.02–0.19	0.006–0.25	0.009–0.18
*P* value	0.032*	0.017*	0.039*	0.030*

Abbreviations: N, number of patients; PPD, periodontal probing depth; PPI, proton pump inhibitors.

^a^ NS/ND—non‐smokers and non‐diabetics.

^b^ NS/ND/NMed—non‐smokers, non‐diabetics, and patients without systemic conditions (see Methods).

* Statistical significance *P* < 0.05.

**Table 2:** Plaque control efficacy among patients using PPIs versus not using PPIs

Plaque scores‐PPI	Plaque scores (NS/ND)^a^	Plaque scores (NS/ND/NMed)^b^
No PPI	1.59 (N = 569)	1.59 (N = 492)
PPI	1.57 (N = 21)	1.51 (N = 18)
Mean difference (no PPI‐ PPI)	0.01	0.07
Chi‐square (*X* ^2^; degrees of freedom, sample size)	0.093 (1590)	0.013 (1510)
*P* value	0.76*	0.91*

Abbreviations: N, number of patients; PPI, proton pump inhibitors.

^a^ NS/ND—non‐smokers and non‐diabetics.

^b^ NS/ND/NMed—non‐smokers, non‐diabetics, and patients without systemic conditions (see Methods).

* Statistical significance *P* < 0.05.

**Table 3:** Mean age of patients using PPIs versus not using PPIs

Age‐PPI	Age ≥18 years (NS/ND)^a^	Age ≥18 years (NS/ND/NMed)^b^
PPI	61.26 (N = 31)	62.39 (N = 28)
No PPI	55.73 (N = 713)	55.79 (N = 621)
Mean difference (PPI–no PPI)	5.53	6.60
95% Confidence interval of mean difference	0.84–10.21	1.65–11.56
*P* value	0.021*	0.009*

Abbreviations: N, number of patients; PPI, proton pump inhibitors.

^a^ NS/ND—non‐smokers and non‐diabetics.

^b^ NS/ND/NMed—non‐smokers, non‐diabetics, and patients without systemic conditions (see Methods).

* Statistical significance *P* < 0.05.

In the NS/ND/NMed group (Table 1), 14% of teeth were associated with probing depths ≥6 mm among 28 PPI users, in contrast to 24% for the 621 non‐PPI using patients (*P* = 0.030). Likewise, 27% of teeth presented with probing depths ≥5 mm among PPI users versus 40% for non‐users (*P* = 0.039). There were no significant differences in mean plaque score levels when comparing PPI users and nonusers (*P* = 0.532; Table 2). The mean age of PPI users (62 years) was greater than that of non‐PPI users (56 years) in the NS/ND/NMed group (*P* = 0.009; Table 3).

To partially compensate for age differences in the PPI and non‐PPI groups, the analysis was repeated after restricting patient age to 30–70 years in an attempt to eliminate patients at the lower and higher limits of the age distribution (Table 4). With age restriction, 14% of teeth were associated with ≥6 mm probing depths among PPI users, in contrast to 25% for non‐PPI users (*P* = 0.017). Similarly, 28% of teeth had ≥5 mm probing depths among PPI users, in contrast to 42% for nonusers. No significant differences in the mean plaque scores of NS/ND using PPIs or not using PPIs (*P* = 0.702) were found (Table 5). With such age restriction, there were no statistically significant differences in the mean age of patients using PPIs or not using PPIs (*P* = 0.091; Table 6).

**Table 4:** Mean percentage of teeth with elevated periodontal probing depths in patients between 30 and 70 years old using PPIs versus not using PPIs

PPD‐PPI	% Of teeth with PPD ≥5 mm in 30–70 years old (NS/ND)^a^	% Of teeth with PPD ≥6 mm in 30–70 years old (NS/ND)^a^	% Of teeth with PPD ≥5 mm in 30–70 years old (NS/ND/NMed)^b^	% Of teeth with PPD ≥6 mm in 30–70 years old (NS/ND/NMed)^b^
No PPI	42.2% (*N* = 611)	25.3% (*N* = 611)	41.4% (*N* = 530)	24.6% (*N* = 530)
PPI	27.8% (*N* = 26)	13.9% (*N* = 26)	27.1% (*N* = 23)	14% (*N* = 23)
Mean difference (no PPI–PPI)	14.4%; 34% decrease	11.4%; 45% decrease	14.3%;34% decrease	10.6%; 43% decrease
95% Confidence interval of mean difference	0.01–0.27	0.02–0.20	0.01–0.28	0.01–0.20
*P* value	0.028*	0.017*	0.039*	0.034*

Abbreviations: N, number of patients; PPD, periodontal probing depth; PPI, proton pump inhibitors.

^a^ NS/ND—non‐smokers and non‐diabetics.

^b^ NS/ND/NMed—non‐smokers, non‐diabetics, and patients without systemic conditions (see Methods).

* Statistical significance *P* < 0.05.

**Table 5:** Plaque scores from patients between 30 and 70 years old using PPIs versus not using PPIs

Plaque scores‐PPI	Plaque scores in 30–70 years old (NS/ND)^a^	Plaque scores in 30–70 years old (NS/ND/NMed)^b^
No PPI	1.58 (*N* = 500)	1.58 (*N* = 428)
PPI	1.54 (*N* = 18)	1.46 (*N* = 15)
Mean difference (no PPI–PPI)	0.04	0.12
Chi‐square (X^2^; degrees of freedom, sample size)	0.156 (1518)	0.003 (1443)
*P* value	0.69*	0.96*

Abbreviations: N, number of patients; PPI, proton pump inhibitors.

^a^ NS/ND—non‐smokers and non‐diabetics.

^b^ NS/ND/NMed—non‐smokers, non‐diabetics, and patients without systemic conditions (see Methods).

* Statistical significance *P* < 0.05.

**Table 6:** Mean age of patients between 30 and 70 years old using PPIs versus not using PPIs

Age‐PPI	30–70 Years old (NS/ND)^a^	30–70 Years old (NS/ND/NMed)^b^
PPI	57.58 (N = 26)	58.48 (N = 23)
No PPI	54.34 (N = 611)	54.57 (N = 530)
Mean difference (PPI–no PPI)	3.24	3.91
95% Confidence interval of mean difference	−0.52 to 6.99	−0.065 to 7.88
*P* value	0.091*	0.054*

Abbreviations: N, number of patients; PPI, proton pump inhibitors.

^a^ NS/ND—non‐smokers and non‐diabetics.

^b^ NS/ND/NMed—non‐smokers, non‐diabetics, and patients without systemic conditions (see Methods).

* Statistical significance *P* < 0.05.

Similarly, after restricting age to 30–70 years among subjects in the NS/ND/NMed group (Table 4), 14% of teeth were associated with ≥6 mm probing depths among patients using PPI versus 25% for patients not using PPIs (*P* = 0.034). Likewise, 27% of teeth were associated with ≥5 mm probing depths in PPI users and 41% in nonusers (*P* = 0.039). No statistically significant differences were seen in the mean plaque score levels in PPI users and nonusers (*P* = 0.350; Table 5), or in the mean age of patients using or not using PPIs (*P* = 0.054; Table 6).

## 4 DISCUSSION

The results suggest that fewer teeth with elevated probing depths occur among patients using PPIs, compared to patients not using PPIs, thus supporting the hypothesis of an association between PPI and periodontal disease. Probing depths ≥5 and ≥6 mm were calculated to assess the effect of using two measures of periodontal disease severity. In all cases, no significant differences in the plaque scores from patients either using or not using PPIs were found, implying that plaque control was not a confounding factor.

Smokers have between twofold and sevenfold increase in risk for having periodontitis or attachment loss compared to nonsmokers (Albandar, 2002). Similarly, diabetes mellitus also is considered to be a major risk factor with an approximate threefold increase, compared with nondiabetic individuals (Preshaw et al., 2012). Other systemic factors such as chemotherapy, systemic steroids, and hormone replacement therapy, as well as non‐thyroid autoimmune diseases such as rheumatoid arthritis and systemic lupus erythematosus, have the potential to affect the severity of periodontal diseases (Marques et al., 2016; Marques & Dib, 2004; Mayer et al., 2013; Scarabelot et al., 2014). Consequently, smokers and diabetics, as well as patients having other potential systemic confounding factors, subsequently were excluded from the analysis. Following such exclusion, the relationship between PPIs and a smaller percentage of elevated pocket depths persisted.

Patients using PPIs tended to be older, having a mean age of 61 years, while nonusers had a mean age of 56 years. Indeed, patients using PPIs are expected to be older, since those medications generally are prescribed for adult patients with GI conditions that become more prevalent over time. Although the age differences between the PPI and non‐PPI groups were statistically significant, it is questionable whether those differences were of clinical significance. Nevertheless, to address this potential source of bias, the analyses were repeated after applying age restrictions to remove possible outliers at the limits of the age range. Following that adjustment, there were no statistically significant differences among patients using or not using PPIs, but significant differences persisted in the percentage of elevated probing depths between patients using, or not using PPIs. Finally, the literature is replete with studies demonstrating that age is a risk factor for periodontal disease (Albandar, 2002). However, the present results indicate that *smaller* probing depths were found in the older PPI population. Therefore, it is less likely that age was a confounding factor in this study, since one might anticipate a greater risk for periodontal disease in the older (PPI) group.

In general, PPIs have been associated with decreased bone mineralization, an increased incidence of osteoporosis, depressed bone metabolism, and an altered microbiome (Jackson et al., 2016; Vestergaard et al., 2006; Wagner, 2018; Yang, 2012). It is tempting to speculate that those effects might combine to decrease the rate of bone resorption which, in a periodontal environment, leads to a decrease in periodontal disease severity. Reports have suggested that PPIs do not favor implant success (Aghaloo et al., 2019; Ursomanno et al., 2020; Wu et al., 2017) but, unlike natural teeth, implants require osseointegration, the periodontal ligament is absent, and trauma to bone is inherent to the surgical implant placement process. Similarly, the reported increase in long bone fractures among PPI users secondary to decreased mineralization (Yang, 2012) might not be directly comparable to periodontal diseases associated with altered inflammatory pathways and microbiologic pathogenesis.

Consequently, the precise mechanism by which PPIs could decrease the severity of periodontal disease remains to be elucidated. Previous studies suggest that PPIs can alter bone homeostasis, influence inflammatory regulation, and affect the GI microbiome (Williams & McColl, 2006).

Mishiro et al., in a randomized controlled trial, found decreased levels of *Neisseria* and *Veillonella* genera in saliva, as well as increased levels of *Leptotrichia* in gingival crevicular fluid (Mishiro et al., 2018). Although the clinical significance of alterations in the bacterial composition has yet to be determined, esomeprazole, a PPI, altered the oral microbiota composition in that study (Mishiro et al., 2018). Consequently, it is conceivable that an altered microbiome might have an effect on periodontal pathogenesis.

Vacuolar H^+^‐ATPase (V‐ATPase) proton pumps in osteoclasts are mainly responsible for creating an acidic environment between the ruffled border of osteoclasts and bone tissue (Prause et al., 2015). The V‐ATPase proton pump is located in the bone‐apposed plasma membrane of the osteoclast where, under acidic environment, lytic enzymes are activated and bone is resorbed during the course of bone remodeling (Costa‐Rodrigues et al., 2013; Prause et al., 2015). Indeed, Prause et al., in an in vitro study, investigated the effect of pantoprazole on a cellular level (Prause et al., 2015). They found pantoprazole to significantly inhibit the ability of human osteoclasts to degrade and absorb bone matrix (Prause et al., 2015). Consequently, it is conceivable that PPIs might provide some protection for a host susceptible to periodontal diseases, where a decrease in osteoclastic activity might lead to a slower rate of bone remodeling or resorption (Costa‐Rodrigues et al., 2013).

PPIs have been associated with potential anti‐inflammatory effects that appear to be independent of their effect on gastric acid secretion (Kedika et al., 2009). PPIs inhibit mononuclear cell chemotaxis and phagocytosis, suppress the inflammatory cell oxidative burst, and inhibit the expression of cell adhesion molecules (Handa et al., 2006). As a result, it is possible that PPIs could affect periodontal pathogenesis and have a possible therapeutic role in reducing the severity of periodontitis (Ali et al., 2009). Potentially, nonsurgical periodontal therapy in conjunction with short‐term use of PPIs might be considered as future adjunctive therapy in a manner similar to the use of NSAIDS and local antimicrobial delivery (Dias et al., 2019). However, additional studies on the underlying mechanism of action of PPIs are indicated prior to recommending the use of those medications as a periodontal treatment modality.

Unmodifiable study limitations included those inherent to a retrospective design included lack of information regarding PPI dosage, duration, and patient compliance, and the use of self‐reported patient medication information. However, the retrospective design also reduced examiner bias, since patient histories and clinical measurements were obtained prior to study initiation. Inclusion of larger and more diverse study populations also would provide additional statistical power and provide information regarding the generalizability the reported results. Accordingly, to more completely characterize the role of PPIs in periodontal pathogenesis, prospective cohort studies, randomized clinical trials, and animal studies currently are planned or underway in our laboratory.

## 5 CONCLUSIONS

The results suggest that use of PPIs is associated with a reduced proportion of teeth with elevated probing depths, implying that PPIs might play a role in reducing the severity of periodontitis. Future prospective studies are indicated to further characterize that association and to assess whether PPIs have the potential to serve as an adjunct in periodontal therapy.

## CONFLICT OF INTEREST

The authors declare no conflicts of interest.

## AUTHOR CONTRIBUTIONS

Lisa M. Yerke and Robert E. Cohen conceived and designed the research project, and were responsible for data collection and analysis. Bhavneet K. Chawla was the main author in writing the manuscript. Lisa M. Yerke and Robert E. Cohen edited the manuscript. All authors approved the final version.

## CLINICAL SIGNIFICANCE

*Scientific rationale for the study*: Proton pump inhibitors (PPIs) are frequently prescribed to reduce gastric acid secretion. PPIs also affect bone metabolism and are associated with gut dysbiosis, but their potential effect on periodontitis has not previously been reported. Consequently, the aim of this study was to measure the effect of PPIs on periodontitis.

*Principal findings*: PPIs are associated with a significantly reduced proportion of teeth with elevated probing depths, suggesting less severe periodontitis among PPI users.

*Practical implications*: PPIs may have a potential therapeutic role in reducing the severity of periodontitis, which warrants additional investigation.

## Data Availability

Data available from the authors upon request.
